# Virtual Reality for Obsessive-Compulsive Disorder: Past and the Future

**DOI:** 10.4306/pi.2009.6.3.115

**Published:** 2009-07-08

**Authors:** Kwanguk Kim, Chan-Hyung Kim, So-Yeon Kim, Daeyoung Roh, Sun I. Kim

**Affiliations:** 1 Department of Biomedical Engineering, Hanyang University, Seoul, Korea.; 2 Department of Psychiatry and Institute of Behavioral Science in Medicine, Yonsei University College of Medicine, Seoul, Korea.; 3 Department of Psychology, University of North Carolina at Chapel Hill, Charlotte, NC, USA.

**Keywords:** Obsessive-compulsive disorder, Virtual reality, Computer, Assessment, Treatment, Cognitive-behavioral therapy

## Abstract

The use of computers, especially for virtual reality (VR), to understand, assess, and treat various mental health problems has been developed for the last decade, including application for phobia, post-traumatic stress disorder, attention deficits, and schizophrenia. However, the number of VR tools addressing obsessive-compulsive disorder (OCD) is still lacking due to the heterogeneous symptoms of OCD and poor understanding of the relationship between VR and OCD. This article reviews the empirical literatures for VR tools in the future, which involve applications for both clinical work and experimental research in this area, including examining symptoms using VR according to OCD patients' individual symptoms, extending OCD research in the VR setting to also study behavioral and physiological correlations of the symptoms, and expanding the use of VR for OCD to cognitive-behavioral intervention.

## Introduction

Obsessive-compulsive disorder (OCD) is a debilitating and chronic mental disorder, with lifetime prevalence rates estimated to be as high as 2.5% in the United States^1^ and 2% worldwide.^2^ People with OCD experience symptoms such as intrusive, unwanted thoughts and ideas that cause an increased amount of anxiety (i.e. obsessions), and intentional, repetitive behaviors that decrease anxiety (i.e. compulsions).^3^ According to Rasmussen and Eisen, the most frequent obsession is 'contamination' and the most frequent compulsion is 'checking' in adults with OCD.^4^ Other obsessions include 'pathological doubt', 'somatic', 'need for symmetry', 'aggressive', and 'sexual' and compulsions include 'washing', 'counting', 'need to ask or confess', 'symmetry and precision', and 'hoarding'.^4^

Current reliable and valid OCD measures can be classified into clinician-version inventories and self-report questionnaires. Clinician-version inventories include the Yale-Brown Obsessive-Compulsive Scale (Y-BOCS)^5^,^6^ and others. Self-report based inventories include the Maudsley Obsessive Compulsive Inventory (MOCI)^7^ and others. These self-report and clinician-rated assessment tools are well established and play a critical role in the assessment of OCD symptoms. However, because such assessment methods are inherently based on retrospective patient reports, they are unlikely to provide exact details regarding the frequency and duration of compulsive behavior. Thus, an assessment tool that includes the use of self-reports and behavioral measures of OCD symptoms would be beneficial as it would provide a more comprehensive assessment of symptoms.

As the case for assessment, effective treatments for OCD are also available, both in psychological and pharmacological.^8^ Yet, people with OCD have a low rate of helpseeking behavior. For example, a study found that only 20% have sought help with a trained mental health professional.^9^ Several researchers have suggested several reasons for this low rate of treatment seeking, including factors common to all mental disorders such as a lack of trained professionals and the prohibitive cost of treatment^10^ and reasons specific to those with OCD, such as reluctance to engage in exposure^10^ and uneasiness to recognize obsessions.^11^ Nevertheless, none of the current methods are sufficient to solve possible problems related to the low rate of help-seeking behavior in patients with OCD. Thus, a new approach that directly responds to those possible reasons is necessary.

Virtual reality (VR) is one of the best candidates as a tool for assessing and treating OCD patients. VR integrates real-time computer graphics, sounds, and other sensory input mechanisms to create a computer generated world with which the user can interact. Because of these features, VR can serve as an alternative, patient-friendly assessment and treatment tool for OCD patients. In fact, it has been concluded that VR has a variety of theoretical advantages for the treatment of several mental problems over other traditional approaches.^12^,^13^ VR-based therapy is based on the principle of exposure similar to traditional approaches. However, VR offers a safer and cost-effective alternative, in some conditions, compared to the traditional approaches, in which vivo exposure is impractical, difficult, and potentially dangerous (e.g. driving phobia) or when the cost of treatment is prohibitive (e.g. flight phobia). Like imaginal exposure, VR exposure takes place in a controlled environment (usually the therapist's office), and patients do not have to be exposed to the real situation in order to provoke a fear response. Moreover, VR exposure may be perceived as safer than *in vivo* exposure since the patient knows that the technology can be switched off at any time. Feeling that they are in control of the VR experience may also serve to increase patients' feelings of self-efficacy. Moreover, the less aversive environment of VR relative to the actual setting may increase the number of patients seeking treatment and decrease attrition rates. VR environments are also flexible, the therapist can readily adapt the environment to individual fear hierarchies. Furthermore, VR can be used to recreate situations that cannot be re-experienced *in vivo* such as a combat situations or, for example, the attack on the World Trade Centre. VR exposure can be used as an alternative to imaginal exposure in such situations because patients do not need to rely on internal imagery or their ability to visualize a situation. Furthermore, in imaginal exposure the therapist has no control over, or even knowledge of, what imagery the patient actually evokes, whereas the therapist controls the stimuli that the patient is being presented with in the virtual environment.

Due to these advantages, VR-based exposure therapy has been developed and applied to the assessment and treatment of various mental problems including the treatment of anxiety disorder^13^ and specific phobias such as the fear of heights,^14^-^16^ fear of flying,^17^-^21^ driving phobia,^22^-^24^ spider phobia,^25^,^26^ social phobia,^27^-^29^ and agoraphobia.^30^,^31^ VR applications have also been developed for the treatment of post-traumatic stress disorder,^32^-^34^ body image disturbance,^35^-^37^ male sexual dysfunction,^38^ attention deficit in children,^39^ and test anxiety.^40^,^41^ Nonetheless, the use of VR for OCD is still in short supply, possibly due to the heterogeneous symptoms of OCD and poor understanding of the link between VR and OCD. To provide a comprehensive understanding of the need and use of VR in OCD, we will review the empirical literature regarding computer-administered OCD research as well as current VR studies for OCD. We will then discuss the limitations and future challenges of VR studies for patients with OCD.

## Utilizing Computers for Assessing and Treating Obsessive-Compulsive Disorder

In the early 1990s, the first computerized assessment for OCD, named Karaepelin, was developed.^42^ Karaepelin used a total 50 natural language questions and 115 reasoning rules to either reach an OCD diagnosis or to suggest one of several differential diagnoses.^43^ A year later, Rosenfeld et al.^44^ introduced and evaluated a computerized version of the Y-BOCS, which is the most widely used measure of OCD symptoms. The authors showed that the computerized version of the Y-BOCS was as good as the clinician-version Y-BOCS for measuring symptoms in OCD. Other adaptations of the Y-BOCS came in the form of computerized system used telephone-based interaction.^45^,^46^

One of the next major advances in the computer-based assessment of OCD was the development of BT STEPS, which combined assessment and treatment into one comprehensive package.^47^ The BT STEPS program has been used in numerous studies and the assessment portion has been completed by 84% of people who started it.^46^ This interactive voice response (IVR) program had the clients list their rituals, the cost of performing them, and the triggers for each ritual.

Also, Herman and Koran^48^ published a study investigating the use of handheld computers for the assessment of OCD symptoms as they occurred in the actual environment. The researchers found moderate agreement between clinician-version Y-BOCS scores and data gathered from the handheld computer, which was possibly due to the small sample size and some methodological difficulties regarding the use of the computers.

The study by Rotge et al.^49^ focused on the checking behavior of OCD. They concentrated on the objective and quantitative measurement of checking activity, which represents the most frequently observed compulsion in OCD. To address this issue, they developed an instrumental task producing repetitive checking in OCD subjects. Fifty OCD subjects and 50 normal volunteers (NV) were administered a delayed matching-to-sample task that offered the unrestricted opportunity to verify the choice made. Response accuracy, number of verifications, and response time, which reflected the degree of uncertainty and doubt, were recorded over 50 consecutive trials. Despite similar levels of performance, patients with OCD demonstrated a greater number of verifications and a longer response time in making a choice before checking than NV. Such behavioral patterns were more pronounced in OCD subjects currently experiencing checking compulsions. This task might be of special relevance for the quantitative assessment of checking behaviors and for determining relationships with cognitive processes.^49^ The earliest published records of computer-based treatment for OCD were performed by Baer et al.,^50^,^51^ who developed a program that ran on a handheld, portable computer to assist in the treatment of clients with OCD. This treatment was called OCCHECK, and it served two purposes: reminding clients of the instructions given during treatment sessions and tracking information about the intensity and frequency of the obsessions and compulsions. The authors reported findings from two case studies in which each client experienced a large reduction in symptom occurrence when using the computer-based treatment, and revealed an increase in symptoms when the computer was removed. Furthermore, clients showed a subsequent decrease in symptoms when the computer was reintroduced. Despite a small number of cases, these publications gave impetus on the development of using computers to treat OCD.

Although the authors did not report any statistical data, a reduction in symptoms of OCD after using BT STEPS was comparable to that of selective serotonin reuptake inhibitors (SSRI), a commonly prescribed medication for OCD patients. In the following year, Greist et al.^52^ reported self-treatment for OCD using the IVR system described previously. Following a baseline period, participants performed a self-assessment and then self-treatment over the course of 12 weeks. A 22-week open period then followed in which participants could still access the system at any time they desired. At the end of the 22-week open period, 61% of the participants rated themselves as either "much improved" or "very much improved".^53^

Computer-based treatments have also been used as an adjunct to behavioral therapy for OCD. Clark et al. provided baseline measures of obsessive and compulsive symptoms, including the Y-BOCS, PI, and Beck Depression Inventory (BDI) to 13 participants with OCD and 10 without.^54^ For all total scores, the participants with OCD had significantly higher scores than non-OCD participants. After the assessment session, all participants attended three sessions of treatment in which they were guided by a computer screen exposed to dirt without washing their hands. Results indicated that participants with OCD displayed significant decrease in both overall levels of depression as measured by the BDI and obsessions and compulsions as measured by PI. Moreover, those participants identified as 'washers' rather than 'checkers' appeared to show more OCD symptom improvement, while checkers showed more depressive symptom improvement. The authors concluded that this program, although not appropriate for stand-alone treatment, might be useful as an introduction to behavior therapy.^54^

In the largest study examining computer-version treatment for OCD to date, Greist et al.^55^ compared behavioral therapy delivered via a computer (BT STEPS) to therapist-guided exposure therapy for OCD to patients in a relaxation control group. Both the BT STEPS and clinician-guided therapies showed significant changes in Y-BOCS scores among OCD patients compared to the relaxation group. Participants in the clinician-guided therapy showed greater improvement in Y-BOCS scores than those in the BT STEPS group. However, other analyses showed no difference between BT STEPS and clinician-guided therapy in terms of a decrease in the number of hours spent on rituals and compulsions in OCD patients.

Overall, a number of researchers have applied a variety of computer-based assessments and treatment tools for OCD patients and demonstrated reliable results using those computerized methods. Researchers also found benefits in using computerized methods for the assessment and treatment of OCD patients over traditional behavioral options. The summary of the previous findings are illustrated in Table 1.

## Utilizing Virtual Reality for Obsessive-Compulsive Disorder

Despite active development and use of computer-based assessments and treatments for OCD patients, the applications of VR for OCD are still somewhat lacking. However, the applications of VR in assessing and treating patients with OCD can be advantageous for several reasons. First, as mentioned previously, VR-based exposure therapies have demonstrated efficacy in patients with other anxieties by reducing patients' anxiety levels during therapy. Second, by quantifying the patterns of behavior within the virtual environment, VR allows precise measurement of performance in a controlled and replicable task.^56^ Third, objective behavioral assessment of OCD in the natural environment is prohibitively difficult, and behavioral assessment tasks (BATs) used to assess OCD symptoms are usually restricted in form to a narrow class of general stimuli that may not be personalized or relevant to any given OCD patient. That is, most BATs used in clinical practice are unlikely to be personally tailored. However, VR-based behavioral assessments are flexible enough to create personally-relevant virtual environments, rather than using a limited number of BATs for all OCD patients. Finally, although self-report and interviewer-rated measures of OCD symptoms have demonstrated sound psychometric properties,^57^,^58^ these assessments are susceptible to multiple forms of bias, which complicate clinical outcome evaluations. VR-based methods can overcome bias-related problems and provide more objective measures of OCD symptoms.

Because of the advantages of using VR on OCD patients, several researchers have developed VR-based tools for assessing patients with OCD. Recently, Kim et al.^59^ developed a VR-based checking behavior assessment for patients with OCD. The authors examined performance on a novel behavioral measure in 30 patients with OCD and 27 matched healthy controls. In the VR assessment, participants navigated virtual environments using a joystick and head-mounted display. The experiment consisted three phases: training, distraction, and the main task. After the training and distraction phases, participants were instructed to freely explore virtual environment as if they were in their natural environments. Primary dependent variables included several indices of frequency and duration of checking behaviors: frequency of checking behavior, gazing at the time during checking behavior, length of trajectory, and checking time. The researchers examined construct validity for the task by comparing the novel behavioral measures with standardized self-report and interviewer-rated measures. Results indicated that the OCD patients demonstrated significantly greater problems with compulsive checking compared to the controls, and performance on the VR task was positively correlated with both self-reported symptoms and interviewerrated measures associated with OCD. This study therefore provides preliminary data to support the use of VR as a possible behavioral measure of compulsive checking behavior in OCD.

As a basic step of exposure response prevention (ERP) for patients with OCD, a preliminary test of a VR anxiety-provoking tool has also been introduced using a sample of participants with OCD.^60^ The experiments were conducted on 33 participants with OCD and 30 healthy controls. Results revealed that those with OCD had significantly higher anxiety in the virtual environment than did healthy controls, and the decreased ratio of anxiety in participants with OCD was also higher than that of healthy controls. Moreover, the degree of anxiety in individuals with OCD was positively correlated with their symptom scores and immersive tendency scores. Hence, these results also suggest the possibility that VR technology has a value as an anxiety-provoking or treatment tool for OCD.

To evaluate the sensitivity of VR tools for targeting OCDs and OCD controls, the comparison between OCD checkers whose major symptoms are checking behavior in their homes and OCD controls who did not have or mildly had checking symptoms was performed using a VR-based assessment (K. Kim, PhD, C-H Kim, MD, PhD, K-R Cha, MD, M. Rosenthal, PhD, J-J Kim, MD, PhD, I. Kim, MD, PhD, S. Kim, PhD, unpublished data). The study involved 31 healthy controls, 17 OCD control patients, and 22 OCD checking patients, and the researchers found that the VR behavioral parameters used in their study appeared to be effective sensitivity assessment measures or indicators for evaluation of compulsive checking behavior among OCD patients. These findings indicate that the behavioral parameter in VR is capable of measuring a range of parameters associated with specific compulsive checking behavior.

## Discussion

The findings from Kim and colleagues^59^ provide the first steps towards using VR as an objective behavioral measure of symptoms in patients with OCD. With appropriately designed study with larger sample size, we believe that VR-based methods may be able to improve the assessment and treatment of OCD, by providing more objective information about the patient's symptoms.

The applying of VR to OCD patients has several significant potential applications in assessing and treating symptoms in OCD patients. Traditionally, symptom assessment was usually carried out with the interviewer and patients with retrospective recalling the past week or month, while sitting in a clinic room. In contrast, VR can provide a standardized assessment of actual symptom occurrences. Recent works involving patients with schizophrenia provide good examples for symptom assessment by VR. According to Freeman's recent report, individuals who experience auditory hallucinations in social situations have reported voices in the virtual train.^61^ As seen in schizophrenia cases, assessing the symptoms of OCD patients using VR will provide a more objective understanding of their symptoms. For example, if we want to determine the symmetry or the arrangement symptom of OCD patients, we can use VR environments such as a desk, street, or refrigerator for eliciting their symptoms and testing their severity.

VR is capable of taking a large range of behavioral parameters associated with patients' symptoms, and this allows researchers to investigate behavioral and physiological correlates of the symptoms. Specifically, it is simpler to record a participant's movement in VR than in the actual environment. Moreover, physiological measures such as heart rate, blood pressure, and skin conductance can be taken concurrently with VR measurements. With phobic patients, Mühlberger et al.^62^ evoked fear responses from patients in VR settings and demonstrated patients' behavioral measures and their physiological symptoms in VR. Moreover, Garau et al.^63^ demonstrated the analysis that VR events do result in sudden change in physiological measure level. Although no existing OCD studies tested correlation between patients' physiological measures and behavioral responses in the VR setting, Kim et al.^59^ demonstrated promising results that VR is a sensitive measure for specific behavioral symptoms in patients with OCD. They demonstrated construct validity by demonstrating significant positive correlations between task performance and both self-reported and clinician-rated measures. Although the percentage of successfully completed tasks was equivalent between OCD patients and HC, the OCD patient group had a higher frequency of compulsive checking behavior, longer gazing time during checking behavior, and longer length of behavioral trajectory in the virtual environments compared to the HC groups. These findings indicate that behavioral tasks using VR are capable of measuring a range of behavioral parameters associated with OCD symptoms. Moreover, the correlation between both task performance and self-reported measures show the possibility of a relationship between patients' behavioral measures and their physiological symptoms. A future study can utilize those behavioral measures to investigate the relationship between physiological responses and behavioral symptoms in OCD patients.

The use of VR with the emerging cognitivebehavioral interventions for psychosis,^64^,^65^ and developing treatments for patients with OCD is one of the ultimate goals of VR application. Although VR has been used as an effective treatments tool for other psychological disorders (e.g., anxiety disorder),^13^ there is no established work using VR to treat symptoms in OCD patients. The only relevant study that exists used VR as an anxiety provoking tool for participants with OCD.^60^ The researchers found that VR was a valuable method for anxiety provocation. This study suggests a potential use of VR for the treatment of OCD. For this, we would like to highlight the importance of using personally relevant virtual environments. There is wide variability in the symptom contents of people with OCD, and therefore, triggering environments will differ. Further research with personally tailored-stimuli in VR settings could provide effective treatment for OCD patients. As a first step we would like to emphasize symptom-based treatment programs like checking-, contamination-, and symmetry-response prevention. The virtual environments developed for social-, claustro-, and agora-phobia provide good examples for future studies.

There are also limitations for the application of VR to assess and treat OCD patients. The main issue to be considered is simulator sickness, which can involve dizziness, nausea, headache, and eyestrain.^66^ Also, it is inadvisable to apply VR environment to individuals who have seizures when watching television or playing video games. However, according to the authors' previous experiments, the most common comments from patients are that they enjoyed the experience and appreciated the opportunity to try a new technology. Researchers who desire to use VR with OCD patients will be able to overcome unwanted side effects, by routinely monitoring specific equipment, the scenarios, and the length of immersion in order to determine the best VR environment for each patient.^67^

In conclusion, despite the fact that the results regarding VR applications for patients with OCD are preliminary, the findings from previous studies showed promising first steps towards using VR as an assessment and treatment for patients with OCD. Moreover, a number of researchers have utilized a variety of computer-based assessments and treatment tools for OCD patients, and demonstrated reliable results using those computerized methods. We believe that VR-based measures may be able to improve the assessment and, ultimately, treatment of OCD after an appropriately designed study that investigates symptom assessment, behavioral and physiological correlates of symptoms, and personally tailored treatment. We think that the use of VR can improve the assessment of OCD in both clinical and research settings, when merging the traditional behavioral research with this novel method.

## Figures and Tables

**TABLE 1:**
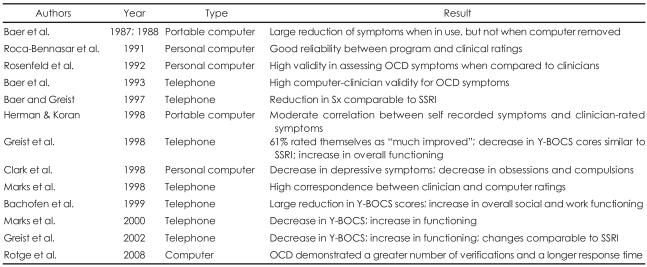
Utilizing computers for OCD42)
